# Just like fireworks in my brain – a Swedish interview study on experiences of emotions in female patients with eating disorders

**DOI:** 10.1186/s40337-021-00371-2

**Published:** 2021-02-17

**Authors:** Suzanne Petersson, Lydia Gullbing, Kent-Inge Perseius

**Affiliations:** 1 Department of Medicine and Optometry, Linnaeus University, Länssjukhuset, Hus 13, plan 7, SE-391 85 Kalmar, Sweden; 2 Division of Rehabilitation, Region Kalmar County, Länssjukhuset, Hus 13, Plan 7, SE-391 85 Kalmar, Sweden; 3 Division of Psychiatry, Region Kalmar County, SE-391 85 Kalmar, Sweden; 4 Department of Caring Science, Linnaeus University, SE-39182 Kalmar, Sweden

**Keywords:** Eating disorders, Emotion regulation, Qualitative interview, Thematic analysis

## Abstract

**Background:**

Patients with eating disorders have reported poorer emotional awareness, more emotional suppression, less use of adaptive emotional regulation strategies, and more use of maladaptive emotional regulation strategies compared to people in healthy control groups.

**Aim:**

To explore experiences of emotions by a transdiagnostic sample of patients with eating disorders.

**Method:**

Nine patients with different eating disorder diagnoses at an eating disorder outpatient clinic in Sweden were interviewed for their thoughts on emotions. The interviews were analyzed with Thematic Analysis.

**Result:**

Four themes were constructed: “Not knowing what one feels”, “Switch off, run away, or hide behind a mask”, “Emotions in a lifelong perspective”, and “Using eating behaviours to regulate emotions”. The patients described uncertainty regarding whether they experienced emotions correctly. They described how they tried to avoid difficult emotions through suppressive strategies and eating disorder behaviour. All described strategies were inefficient and all emotions were experienced as problematic, even joy. Since joy was used as a mask, the real experience of happiness was lost and mourned.

**Conclusion:**

All kinds of emotions were considered problematic to experience, but shame, fear, and sadness were considered worst. It is difficult to know if the emotional difficulties preceded an eating disorder, however such difficulties may have increased as a result of the eating disorder.

## Plain English summary

Previous studies on patients with eating disorders have described problems with emotional management, for example: lower emotional awareness, greater emotional suppression, and difficulties in using adaptive emotional regulatory strategies compared to people without eating disorders. In the present study we explored experiences of emotions described by nine women treated for different types of eating disorders. We interviewed patients at a clinic for eating disorder treatment in Sweden. We organized their narratives into four themes; “Not knowing what one feels”, “Switch off, run away, or hide behind a mask”, “Emotions in a lifespan perspective”, and “Using eating behaviours to regulate emotions”. All patients save one described uncertainty regarding whether they experienced their emotions correctly. The patients described how they tried to avoid difficult emotions through suppressive strategies and eating disorder behaviour. All strategies mentioned were inefficient and all emotions were experienced as problematic, even joy. Joy was used as a mask towards others, and therefore their experiences of happiness were lost and mourned. It is difficult to know if the patients had had these difficulties in managing emotions prior to the development of their eating disorders, or if they were developed as a result of them.

## Introduction

Emotions work as guides for e.g. motivation, decision-making, goal setting, evaluation, action, learning enhancement, perspective evaluation, and as promotors of attachment [1]. It is necessary and functional to be able to both act upon and inhibit emotions to behave beneficially [1]. Emotion regulation comprises emotion awareness, understanding and acceptance of emotions, and the ability to control and manage negatively experienced emotions [2–4].

It has recently been shown that emotion dysregulation is a transdiagnostic characteristic of eating disorders (ED) [5, 6], with a tendency to be lower in patients with restrictive anorexia nervosa (AN) compared to patients with bulimia nervosa (BN), binge eating disorder (BED), or AN purging sub-type [7]. A greater degree of emotion dysregulation has been shown to correlate to more severe ED symptomatology [8]. It has even been suggested that deficits in emotion regulation are predisposing factors for the development of ED [6, 8, 9]. Patients with ED have reported poorer emotional awareness, emotional suppression, less use of adaptive emotional regulation strategies, and greater use of maladaptive emotional regulation strategies compared to people in healthy control groups [6, 10–15].

It has also been proposed that patients with ED have features of alexithymia [16, 17]. Alexithymia is defined as an inability to define or describe emotions (Greek: a = lack, lexis = word, thymos = emotions) [18]. In contrast to alexithymia, emotional repression is “associated with the exclusion from conscious awareness of specific conflicts and associated emotions” [[19]: p. 493]. In other words, alexithymia is the inability to express emotions, while repressed emotions are emotionally avoided. It is also possible that people with ED can acknowledge emotions, but are uncertain whether they are allowed to do so [20, 21]. However, it has been shown that alexithymia in patients with ED seems to be correlated to comorbid depression, anxiety, or state of mood rather than to ED per se [22, 23]. It has been debated whether an ED causes elevated levels of emotion dysregulation in patients with long-term ED [24]. Effects of starvation and deficient nutritional status has also been suggested to explain emotion dysregulation in patients with AN [25]. Exploring alexithymia/suppression of emotions is complicated, since there is a contradiction that someone with such difficulties can manage to fill out self-assessments on this topic [21]. Regardless of the causes of emotion dysregulation, patients’ subjective narratives on their experiences of emotions and emotion regulation are important as a complement to self-assessment research. Patients’ narratives have been described previously and most interview studies have been conducted with patients with AN [e.g. [20, 26–28]]. For example, in an interview study with patients in an in-patient unit participants described difficulties in recognition of emotions as well as experiences of discomfort when expressing negative emotions [27]. Another interview study explored and compared in-patients with ANs’ views of their emotional processing with their parents’ and clinicians’ views [26]. Fox’s [20] interview study of 11 female patients with AN showed poor meta-emotional and emotion expression skills. Notable is that in all interview studies mentioned here patients were able to reflect upon and talk about their emotions.

Difficulties in expressing emotions, lack of adequate skills to handle feelings, or not having access to emotions due to suppression, deprive individuals of opportunities to make positive long-term decisions according to valued goals, complicate relationships, and in the long run quash opportunities for greater well-being. Therefore, in connection with a research project aiming to evaluate an intervention addressing emotion regulation among a transdiagnostic sample of patients with ED, the opportunity was seized to interview some of the participants about their experiences of emotions. The aim of this study was thus to explore experiences of emotions as narrated by women with different eating disorders at an out-patient clinic in Sweden.

## Method

### Study setting

The study was conducted during 2018–2019 at a youth/adult, integrated psychiatric outpatient clinic, the AnorexiBulimiCenter (ABC), specializing in the treatment of ED in southern Sweden. The clinic receives approximately 150 new patients yearly. Diagnostic evaluation was performed in a clinical interview using the 36-item version of the Eating Disorder Examination Questionnaire (EDEQ) [29] and the Structured Eating Disorder Interview (SEDI) [30].

### Participants and the affect school

All adult patients (age >/= 18 years) at the clinic were asked to participate in an evaluation study of the Affect School. The Affect School (AS) originates from the Department of Psychology at Umeå University, Sweden. The initial aim was to develop treatment for psychosomatic problems e.g. pain. The AS is a learning process, not a therapy [31]. The method is based on the affect theories developed by Tomkins, Nathanson, and Ekman [31]. The intervention consists of an eight-session group treatment with two-hour weekly sessions [31, 32]. Participants receive handouts at the beginning of each session to follow the educational part of the session. All sessions share the same approach: initially 30 min’ psychoeducation on one affect, followed by reflections, a short break, and then followed by a discussion on the specific affect. Every group participant is encouraged to talk about an incident that has triggered this affect for them, and is eventually followed by a longer reflection on the subject [32]. The sessions were led by two licensed psychologists.

Participants were randomized to participate in the intervention or to a control group. All patients who accepted participation were women, ages ranging from 18 to 51 (*n* = 46, mean = 28.9, Median = 25.5). The sample in the RCT was representative for the adult patient population at the clinic regarding ages and frequency of different ED diagnoses, although approximately 2 % of the patient population at the clinic at the time of the study were men.

After completed participation in an Affect School intervention, patients from the six intervention groups were asked by the AS leaders if they wanted to participate in an interview study. Nine patients from five of the groups agreed, ages ranging from 19 to 43 (mean = 26.6, Median = 25.0). The distribution of the DSM-5 ED diagnosis was: two participants with AN, one with BN, and six participants diagnosed with Other Specified Feeding or Eating Disorder (including five participants diagnosed with atypical AN, and one with a low frequency BN). Three participants reported having earned a university degree, one was a university student, four had completed grammar school, and one had finished elementary school. None were unemployed, but four were on sick leave at the time.

### Data collection

Data was collected in semi-structured interviews. The interviews were based on a guide constructed by the authors. The interview started with wider questions on the personal experience of the intervention. Then the questions became more specific focusing on experiences of emotions. The latter part of the interview also included questions about possible new knowledge due to the intervention, affect awareness, and connections between emotions and ED: What roles do emotions play in your life? What is your relationship to emotions? How do you cope with emotions? Have your strategies to cope with emotions changed in any way (after affect school)? Do emotions have significance in relation to your eating disorder? Interviews, audio recorded at the treatment unit, were between 33 and 52 min long. The first author conducted all but one interviews.

### Data analysis

Data was analyzed by Thematic Analysis (TA) [33]. Thematic Analysis is an experiential and theoretically flexible method compatible with certain paradigms within psychology. Themes can be larger or smaller regarding item content. The method is useful for reporting both the reality of participants and penetrating this reality [33].

Data was transcribed verbatim by the first and second authors. The analysis was performed in six steps. Text analyses were conducted using the software program NVivo.12 [34] and then analyzed as follows:

1. The first and second authors individually read and reread the transcripts several times.
2. Sentences and parts of sentences were recognized as initial codes. Correspondence between the coders was intense during this process.
3. The first and second authors then reworked the material together in an inductive process and agreed on seven preliminary themes.
4. All authors met and discussed the codes and preliminary themes.
5. The last author then separately reviewed the codes and abstracted them into six preliminary themes.
6. The first and last author met and reviewed the material once again agreeing on four final themes, which were found consistent with the data.

In order to assure trustworthiness, the first and second authors kept self-reflecting diaries during the research process. The diaries began with analyses of the authors’ pre-understanding stemming from personal life experiences, work as clinical psychologists, and from the research literature [35, 36]. The first author had been working with patients with ED for 20 years, the second for 1 year, while the third author had extensive experience of working in psychiatric care but not with ED patients.

## Results

The following four themes were revealed in the interviews:

1. *Not knowing what one feels*
2. *Switch off, run away, or hide behind a mask*
3. *Emotions in a lifelong perspective*
4. *Using eating behaviours to regulate emotions*

### Not knowing what one feels

All participants but one described difficulties in distinguishing emotions, which resulted in either chaos and anxiety or confusion concerning which feeling was experienced in different situations. They described anxiety as a state where everything seemed to “fuse”, or as a “cloud of anxiety”. The experience of not knowing the real emotion or not understanding the inner emotional experiences was described as difficult. Only one participant stated that she “*could differentiate (her) emotions pretty well*” (No 40).

“*I find it extremely difficult to distinguish feelings. I think it’s very hard to know what you’re really feeling*” (No 7).

“*And then everything just becomes a cloud of anxiety. It’s as if everything melts together and you don’t really understand what’s what, and then everything gets even more difficult*” (No 9).

“*At times everything’s just chaos in my head and I don’t really know what I feel. (..) Then my inner self just stares, nothing works, it all somehow freezes up”. (…*) *“I usually try to describe it as (…*), *as I usually say like fireworks in my brain. Because a thought pops up, then becomes two thoughts (…), and then just sort of continues. I can’t stop it. So sometimes I wish I had an off switch in my brain*” (No 23).

### Switch off, run away, or hide behind a mask

Experiencing one’s emotions was described as tiresome and intimidating, thus switching them off was chosen in order to protect oneself. “Switching off” is described as a common and conscious choice to avoid fear or other painful feelings:

“*I often turn off what I feel and shut myself in*” (No 12)“*I’m the kind of person that can easily switch off (…), something’s wrong but I don’t know what*” (No 40).

“*I’ve shut off, or I’ve wanted to shut down in some way, so I focused on doing things constantly so I wouldn’t have to feel all the time*” (No 41).

Another metaphor for emotional avoidance was “running away”:

“*You don’t want to feel (anything), you try to run from it and push everything away* (…) *I’m more afraid of doing something I shouldn’t do. And then it’s easier to push away the anxiety and just keep it at a distance, but you get tired of avoiding feelings as well*” (No 9).

“*when I felt there was too much in my head, when things got too stirred up, I resorted to what I usually resort to ( …), I go out for a run” (*No 4*).*

Looking good in the eyes of others through excellent performance, compliance, and obliging (others?) were common descriptions, and associated with feigned joy and switched off feelings. Performance was mostly related to kindred persons, but in a lesser degree also directly to “please” the ED. Showing consideration, appearing happy, and displaying competence to others, or skipping meals and exercising extensively, served both as a way of reassuring others (including the ED), not bothering or burdening others, and escaping emotions. Experiences of “faking joy”, “putting on a mask” or “acting like a robot” were expressed by participants. Sometimes emotions had been repressed or used as a mask outwardly for so long that it was hard to remember how it used to be, and thus difficult to experience naturally:

*“I thought I was some sort of perfect person that didn’t get angry and didn’t show that I was sad, and went around thinking I was happy since I had faked it for so long”*(No 4).

“*It gets easy to put on that mask and pretend to be happy all the time, I guess (…) I walked around like a robot and just did everything while I helped out around the house and carried on. I was probably scared, I was probably really scared*” (No 4).

These strategies had a price in the long run. Even pleasant emotions tended to disappear, and thus complicated the possibility of knowing how real happiness feels.

“*I’m so switched off and so used to going around and pretending to be happy all the time, that even joy becomes so charged since I get so I don’t really know when I’m really happy*”(No 4).

Trying to spare others by hiding behind a mask could also obstruct important relationships, and prevent others from understanding and thus providing support:

*“when I just switch off (my feelings) the whole time it’s not so fair to others either ( …), maybe they get angry “why don’t you show anything?”* (No 12).

“*they deserve to know, too ( …), they really have no idea about what’s going on inside (me)”(*No 30*).*

### Emotions in a lifelong perspective

All kinds of emotions were considered difficult to experience, but shame, fear, and sadness were considered the worst, although it was difficult to keep them apart at times.

“*sadness is always so close at hand and I’m often sad ( …) when I’ve been sad I’ve probably actually been very scared”* (No 4).

Participants explained their emotions in a conscious way, and despite their struggle to avoid them they seemed to be able to recognise different emotions.

*“Sometimes it feels like I’m ashamed of nearly everything”* (No 23)*.*

*“I feel shame for so many different things (that) it gets to the point I get stuck in it, that I feel shame more than with those typical eating disorder things like the body or weight or when you eat and so on”* (No 4)*.*

Showing the environment and significant others a happy and competent facade and not being detected for the true person within evoked sorrow:

“*(I am) sorry that no one saw the person behind (the performance)* (No 4).

While all participants talked about shame, fear, and sadness as troublesome emotions, six participants also talked about anger. While the narratives were otherwise coherent, they differed when it came to this emotion. One of them said that she almost never had experienced anger, that this emotion was unimportant to her, while two reported difficulties in expressing anger due to fears of being wrong or fearing rejection from others. One specific reason for anger was the same as for sadness in the example above, anger over not being prevented from continuing the ED. Some felt that they experienced such strong feelings of anger that it was sometimes problematic, although they also considered expressions of anger important.

“*You can’t just let go if you’re at work, then you have to try to withdraw until you’ve calmed down. But if I’m at home and no one sees me, then I can cry, scream, curse, hit something or something like that*” (No 23).

“*I guess it’s better to be angry and then it’s over, than never to be angry so that it boils over and you get so angry you do something stupid*” (No 7).

However, beside avoidance of negative emotions, difficulties in experiencing curiosity and joy were also described. Some participants considered joy to be the easiest emotion to handle; as long as you can still feel it. Joy was sometimes related to wearing a mask towards others, acting happy as described in the first theme above, and in that case joy could be lost. Participants remembered themselves as quite easygoing and happy when they were young children. Simple and pure joy was lost during life, something that was, as a contrast to other emotions, mostly noticed when lacking.

*“then it’s a bit tough if you feel no joy (...) then it’s hard if you really have to SEARCH (for it) (…) if you haven’t felt happy for a while then it feels like something’s missing, it’s not like you ‘well now I haven’t been angry for a while, so now I’ve to get a little angry’*(No 40).

“*I’ve missed (joy) for most of my life*” (No 41).

Losing joy, spontaneity, and interest was a price or a side-effect for remaining in an ED. Something lost, sometimes mourned, and although this loss could have been a motivation to leave the ED it was not motivation enough.

### Using eating disorder behaviours to regulate emotions

Eating disorder symptoms were described as regulators of unwelcomed emotions, and were used as emotional controls or an escape route from aversive experiences. Behaviours like over-eating, starving, and exercising prevented emotions and anxiety. The ED was described as a small and safe place.

“*it’s easier when you can flee to and be*” *within” the eating disorder, it’s easier when I can reduce my world to this*” (No 4).

“*the eating disorder has been a kind of control, a (way) to withhold feelings, I suppose*” (No 41).

Different emotions were related as associated with ED behaviours, as exemplified below:

*“Sorrow is probably, for me, (that which) strikes back on the eating”* (No 41)*.*

“*The eating disorder is a way to handle anxiety and depression*” (No 4).

*“Shame triggers eating disorder symptoms. I’ve thought about not eating because I’ve felt some feelings and exercised to dampen everything*” (No 12).

“*Disgust is associated with the eating disorder*” (No 4).

Eating disorder symptoms were also described as a help to numb emotions and stay focused. The downside of using ED behaviour to escape from emotions is that the ED does not just make the world narrower and safer but blurs personal experiences with thoughts and emotions that are controlled by the ED:

“*that’s what makes it difficult sometimes, what I feel and what the eating disorder feels, or what it means*” (No 41).

## Discussion

Participants in the study expressed difficulties in differentiating their emotions. They described difficulties regarding what they felt, and doubted whether their experienced emotions were actually correct. This uncertainty was described by some participants to be partly due to the ED having taken over their emotional systems. The ED itself caused anxiety and aversive emotions, which made it harder to abandon ED behaviours. Difficulties with emotional regulation are regarded as concerns about both the ability to understand and modulate emotional experiences and expressions [3, 4]. However, there may be difficulties experiencing/expressing certain emotions but not others. Ego-dystonic emotions might be lost due to linkage with negative experiences and will be perceived as self-destructive (e.g. anger), while other emotions will develop without such devastating links [20, 21]. Patients’ insecurity and self-doubt regarding their capability to recognize emotions correctly was shown by Parling, Mortazavi, & Ghaderi [23], who found that the patients *believed* in such difficulties although they were as capable of identifying and describing emotions as a comparison group of females with no ED symptomatology.

When experiencing chaos and overwhelming thoughts and emotions, it is understandable that escaping from emotions was considered a solution by participants. Emotion suppression seems to occur frequently in ED [e.g. [11, 37]]. When using suppression as emotional regulation the experience of the emotion remains, while the emotional expression is reduced. The strategy of suppression is thus inefficient and their arousal tends to increase over time [11]. Participants in the present study described their escape from emotions by “switching off” or “running away from” them or, especially in relation to others, “hiding behind a mask”.

Despite avoidant behaviours, participants struggled with aversive emotions, which could be explained by the insufficiency of their suppression. Shame was generally experienced as troublesome and was associated with many areas, such as shame over one’s entire person, body and soul, and shame over one’s ED problems. Fear was also connected to life itself, and especially a life without an ED. The narratives regarding ED-related fear is in line with findings in other interview studies on patients with AN [20]. Sadness was connected mainly to loss of life due to years of ED, and sometimes to not being seen by significant others. However, displaying sadness is often experienced as weakness [20] and thus difficult to share with others, with the risk of this negative spiral leading to further alienation from others. Narratives of anger and joy were more divergent among participants. Joy was described as lost and mourned while anger was repressed or expressed, with an uncertainty as to whether this was approved or necessary. Thus, participants in the present study spoke of difficulties in emotional differentiation and how they sought to escape painful emotions on the one hand, and how they recognized and discriminated aversive emotions on the other. They also presented different behaviours to escape from feelings of shame, fear, or anger, and the emotions that were mainly handled by eating disorder behaviours/symptoms. In this regard their narratives seemed paradoxical: participants explained their emotions in conscious ways, and despite their struggle to avoid emotions, they seemed to be able to recognize and discriminate between them, at least in a long-term perspective. Perhaps this could be explained by an uncertainty regarding their ability to know what they felt rather than a disability [12, 23], as an effect of suppression [11], or a sign of fear of expressing aversive emotions, rather than not experiencing the same [20, 21]. There is a difference between repressing/avoiding emotions in the situation and a disability to recognize the same [19, 21]. However, participants’ repressed emotions, expressed as “clouds of anxiety” or emotional “chaos”, could perhaps be recognized after completion of treatment with perspective and with more access to their meta-cognitive structures (i.e. a reduction of beliefs about a danger of experiencing and expressing their emotions). This may be achieved through a treatment that addresses and re-constructs such negative beliefs i.e. CBT-E which is the major treatment for EDs at the clinic [38], or through any treatment focused on developing meta-emotional skills, preferably in interaction with others [20].

Emotional avoidance is common in patients with ED, and ED behaviour has been considered to function by adjusting emotions [e.g. [39, 40]]. Experiential avoidance has shown to mediate the relationship between negative emotions and emotional eating [41] and avoidance of emotions has been associated with different ED diagnoses [6]. Avoiding emotions serves several purposes, e.g. protection of relationships with significant others [20] and treatment staff [27], protection of oneself against appearing weak, and thus a possible target of attack for others [26]. Anger in particular, but also sadness and disgust, have been found to be aversive, and even, dangerous emotions [20, 26, 27]. The function of ED were described by participants in this study as ways to mute aversive emotions.

Shutting down emotions hampers possibilities to satisfy needs (fear/sadness), setting limits on others to reduce the risk of being offended, overrun or similar. At the same time it leads to loss of energizing emotions like curiosity/interest or joy/happiness [1]. Thus, when the emotional system is avoided the emotional function will be disabled, and personal needs will not be met. Another negative side effect of turning off one’s emotions concerned communication with significant others and social support thus being prevented.

The question remains: are the difficulties in knowing one’s feelings primary or do they emerge from the ED and/or other emotional repressive behaviours? In other words; are emotions transformed into “fireworks in the brain” by the ED or do these “fireworks” precede the ED? This can best be answered from a retrospective view by individuals having an ED or, even better, having recovered from one. Participants speak about lost feelings e.g. joy and interest, which indicates former emotional abilities, which in turn could include other emotions as well. This indicates that problems with emotional regulation has at least been increased by the ED.

### Methodological issues

Our interest was in how the patients themselves described their reflections of emotions. The authors were aware of, and considered the risk for, confusion between research and therapy when carrying out qualitative interviews [42]. This is especially important, as one of the interviewers also worked as a therapist at the clinic where the interviews took place, and was familiar with ED and therapeutic interventions related to the treatment of ED [36]. The interviewers had no therapeutic/treatment relationship with the participants in the study. One interviewer and the third author, had not worked with patients with ED and thus provided the perspective of the “naïve inquirer”. A bias that may have occurred is that some narratives may have been guided by participants’ wish for socially desired behaviour. Furthermore, it could be argued that the sample was biased whereby we interviewed patients after an intervention focusing on emotions and emotion regulation. However, we do not solely regard that as bias, rather as a consciously made choice in maintaining a purposeful sample [35]. The AS confirms that it is allowed to experience and express emotions, which for some participants, may not have been experienced before the intervention, and thus made it possible to talk about negative emotions as well. The answer of the interview question on whether their strategies to cope with emotions had changed after attending the AS was an experience of permission to express emotions and some patients had started to test this to a small extent. However, they also described that they did not experience that this had made them feel better or changed their behaviour.

Although our sample consisted of women of differing ages and ED diagnoses to represent clinical reality, it was limited to ethnically Swedish women. As the male patients at the clinic declined to participate in the RCT study the male perspective is lacking, which is a shortcoming. Participants were 19–43 years old, and thus the voices of the population under 18 were unheard. About 40% of the patients at the clinic were under 18 years of age at the time for this study and a hypothesis would be that younger aged individuals generally have greater difficulties when it comes to emotion regulation. Participants in this study had different ED diagnoses, and it is possible that a group of patients with restrictive AN also would have reported more difficulties in emotion recognition [12, 43].

There may also be objections to interviews as a research method. It has been argued that it is impossible to gain neutral and natural knowledge, since researchers always color results with pre-understanding [44]. The researchers in this study have tried to deal with this problem by using and communicating reflexivity [36].

## Conclusion

The present interview study aimed to explore experiences of emotions in patients with ED. The women in the study described uncertainty regarding whether they experienced emotions correctly, which is consistent with previous research [12, 35]. Experiencing one’s emotions was described as tiresome and intimidating. All kinds of emotions were considered problematic to experience, but shame, fear, and sadness were considered worst. Participants described how they tried to avoid difficult emotions through suppressive strategies such as “switching off” or “running away from” them or, especially in relation to others, “hiding behind a mask”. Difficult emotions were mainly handled by ED behaviours, e.g. over-eating, starving, and exercising used as prevention against emotions and anxiety. One problem with ED as a strategy was that it tended to blur feelings thus making situations more difficult to cope with. At the same time the ED tended to create other negative thoughts and emotions. All mentioned strategies were inefficient and all kinds of emotions were experienced as problematic, even joy. Since joy was used as a mask, the real experiences of happiness were lost and mourned. On the one hand participants described difficulties in emotional differentiation, but on the other they recognized and discriminated toward aversive emotions. The fact that participants spoke of lost feelings like joy and interest, indicated former (pre-ED?) emotional abilities. It is difficult to know if the emotional difficulties preceded the ED, but the emotional regulation problems seem at least to have increased because of the ED.

### Clinical implications

Problems with emotion regulation is common in patients with ED and interfere with recognition of personal needs, relationships, and communication with others. Necessary information and social support thus get lost. Regardless of whether these difficulties exist before the development of an ED or, in whole or part, are results of the ED, it is important to work with emotions in ED treatment. The problems appear to be at different levels; in some cases patients have access to their emotions but are uncertain whether their emotions are correct, in some cases patients have difficulties in sorting emotions that stem from the ED from emotions stemming from “themselves”, and in other cases the emotional function perhaps once got lost during their lifetime. One important therapeutic task is to explore how individual emotional processing works, and from that, help the patient to develop an emotional repertoire.

## Data Availability

The raw, anonymized interview data can be accessed by contacting the authors.

## Abbreviations

ABC: AnorexiBulimiCenter (psychiatric outpatient clinic for treatment of eating disorders in Sweden)
AN: Anorexia nervosa
BN: Bulimia nervosa
BED: Binge eating disorder
ED: Eating disorder
EDEQ: The eating disorder examination questionnaire
SEDI: The structured eating disorder interview
TA: Thematic analysis
